# Early transcriptional alteration of histone deacetylases in a murine model of doxorubicin-induced cardiomyopathy

**DOI:** 10.1371/journal.pone.0180571

**Published:** 2017-06-29

**Authors:** Izabela Piotrowska, Mark Isalan, Michal Mielcarek

**Affiliations:** 1 Hatter Cardiovascular Institute, University College London, London, United Kingdom; 2 Department of Life Sciences, Imperial College London, London, United Kingdom; 3 Department of Epidemiology of Rare Diseases and Neuroepidemiology, University of Medical Sciences, Poznan, Poland; Universidad de Buenos Aires, ARGENTINA

## Abstract

Doxorubicin is a potent chemotherapeutic agent that is widely-used to treat a variety of cancers but causes acute and chronic cardiac injury, severely limiting its use. Clinically, the acute side effects of doxorubicin are mostly manageable, whereas the delayed consequences can lead to life-threatening heart failure, even decades after cancer treatment. The cardiotoxicity of doxorubicin is subject to a critical cumulative dose and so dosage limitation is considered to be the best way to reduce these effects. Hence, a number of studies have defined a “safe dose” of the drug, both in animal models and clinical settings, with the aim of avoiding long-term cardiac effects. Here we show that a dose generally considered as safe in a mouse model can induce harmful changes in the myocardium, as early as 2 weeks after infusion. The adverse changes include the development of fibrotic lesions, disarray of cardiomyocytes and a major transcription dysregulation. Importantly, low-dose doxorubicin caused specific changes in the transcriptional profile of several histone deacetylases (HDACs) which are epigenetic regulators of cardiac remodelling. This suggests that cardioprotective therapies, aimed at modulating HDACs during doxorubicin treatment, deserve further exploration.

## Introduction

Cardiac remodelling occurs in response to many pathological and toxic stimuli, including genetic abnormalities and chronic administration of cardiotoxic small molecules. Anthracyclines like doxorubicin (adriamycin) are widely used anticancer drugs and constitute part of the standard chemotherapeutical regime for a broad spectrum of malignancies, due to their high effectiveness [1]. They cause, however, both acute and chronic dose-dependent cardiac injury, which severely restricts their use [2]. Although acute doxorubicin-induced cardiotoxicity is mostly manageable in clinical settings, delayed, life-threatening, anthracycline-associated heart failure can appear even decades after cancer treatment. Over the last decades, a number of groups developed mouse models that mimic doxorubicin induced cardiomyopathy. It has been widely demonstrated that doxorubicin causes cell death of cardiomyocytes followed by the appearance of interstitial fibrosis that, on a physiological level, is manifested by a reduction of the ventricular ejection fraction and contractile function (for a review see [3,4]).

The major strategy to reduce the risk of delayed drug-associated cardiomyopathy is to apply dose limitation [5]. In humans, oncologists most often limit the cumulative dose of doxorubicin to 400–450 mg/m^2^, although it is known that a certain degree of myocardial tissue damage may occur even at dosages that are significantly lower than this maximal tolerated dose [6]. Another widely-used strategy is the administration of doxorubicin by continuous infusion; this is believed to reduce the risk of doxorubicin-induced heart failure, despite the fact that it has never been proven to be effective in long-term studies of cancer survivors. The aim is to reduce peak plasma doxorubicin levels, thus limiting the exposure of heart tissue to high concentrations of the drug [7].

Despite the existence of many theories behind the molecular mechanisms of doxorubicin cardiotoxicity, and over 40 years of intensive studies, there are still no effective cardioprotective interventions against anthracycline-induced cardiac injury [3]. There is growing evidence that the pathological processes that lead to heart malfunction and failure are caused by a cascade of rapid post-translational modifications, governed by a powerful epigenetic mechanism [8]. This is likely mediated by a group of enzymes known as histone deacetylases (HDACs) that play a crucial role in histone or protein deacetylation and, consequently, control global gene expression. Mammalian HDACs are a family of 18 proteins, divided into four groups based on structural and functional similarities: class I (HDACs: 1, 2, 3, 8), class IIa (HDACs: 4, 5, 7, 9), class IIb (HDACs: 6, 10), class III (sirtuins 1–7) and class IV (HDAC11 is the sole member); for a review, see [9]. By following the transcriptional signature of *Hdacs* upon chronic treatment with doxorubicin, we sought to provide insights into the subsequent transcriptional changes in the diseased heart.

In this study, using a well-established mouse model of chronic doxorubicin infusion, we show that a dose of doxorubicin that is generally considered as “safe” [10] can induce adverse changes in the myocardium, as soon as 2 weeks after the start of continuous infusion. The adverse cardiac remodelling was associated with morphological features, including a disarray of cardiomyocytes and fibrotic lesions. The transcriptome showed evidence of dysregulation with striking changes in the HDAC genes. These transcriptional changes in major epigenetic regulators are not only molecular markers of cardiac pathological remodelling, but are potential targets for therapeutic intervention, to reduce doxorubicin toxicity.

## Materials and methods

### Mouse maintenance

CBA x C57BL/6 F1 females (wild type mice) were purchased from Charles River. All animals had unlimited access to water and breeding chow (Special Diet Services, Witham, UK), and housing conditions and environmental enrichment were as previously described [11]. Mice were subjected to a 12 h light/dark cycle. All methods related to experimental procedures performed on animals were conducted under a project license from the Home Office, UK, accordingly to guidelines under the Animals (Scientific Procedures) Act 1986 and were approved by an ethical committee at Imperial College London. Experimental groups included the wild type mice (females only) at 10 weeks of age (n = 6).

### Chronic treatment with doxorubicin

Doxorubicin (Sigma) was freshly prepared and diluted in PBS (Sigma). Mini osmotic pumps (Alzet pumps Model 2002, Charles River) were loaded with 200 μl of either vehicle (PBS), or doxorubicin at a dose of 15 μg/g of body weight, allowing diffusion at 0.5 μl/hour, for a period of 14 days. In order to implant mini osmotic pumps, animals were initially anesthetized with 5% isoflurane, and then anaesthesia was maintained at ~1.5% isoflurane throughout the surgical procedure. Alzet pumps were implanted subcutaneously onto the back of each mouse [12]. After 14 days, the mice were culled and their hearts taken for further analysis. Body weight was measured at the beginning and the end of the trial.

### RNA extraction and Taqman real-time PCR expression analysis

Total RNA from whole hearts was extracted with the mini-RNA kit (Qiagen), according to the manufacturer’s instructions. The reverse transcription reaction (RT) was performed using MMLV superscript reverse transcriptase (Invitrogen) and random hexamers (Operon) [12]. All Taqman qPCR reactions were performed using the LightCycler® 480 Instrument (Roche). Estimation of mRNA copy number was determined in triplicate for each RNA sample by comparison to the geometric mean of three endogenous housekeeping genes, *Rpl13a*, *Canx* and *Gapdh* (Primer Design). Primer and probe sets for genes of interest were purchased from Primer Design or ABI. Primers for transcripts of *Hdac*s were previously described [13].

### Immunohistochemistry and confocal microscopy

For immunohistochemistry, hearts were snap frozen in liquid nitrogen, prior to embedding in OCT. 6–10 μm sections were cut using a cryostat (Leica), air dried and fixed in 4% PFA, (paraformaldehyde) in PBS or in acetone, at -20^°^C for 15 min, followed by washing in 0.1% PBS-Triton X-100 [14]. Blocking was achieved by incubation with 5% BSA-C (Aurion) in 0.1% PBS-Triton X-100, for at least 30 min at RT. Immunolabelling with primary antibodies (anti-collagen VI 1 in 100 (600-401-108-05, Rockland); anti-vinculin 1 in 200 (V4505, Sigma) was performed in 0.1% PBS-Triton X-100, 1% BSA-C, overnight in a humidified box at 4^°^C. Sections were washed 3 times in PBS, incubated for 60 min at RT in a dark box, with an anti-rabbit secondary antibody (FITC Invitrogen, 1:1000 in PBS), washed 3 times in PBS and counterstained with phalloidin (Sigma) and DAPI or draq5 (Invitrogen). Sections were mounted in Vectashield mounting medium (Vector Laboratories). Sections were examined using the Leica TCS SP4 laser scanning confocal microscope and analysed with Leica Application Suite (LAS) v5 (Leica Microsystems, Heidelberg, Germany).

### Protein extraction, western blotting and antibodies

Protein lysates from heart tissues were homogenized in RIPA buffer (1% (v/v) NP-40, 0.5% (v/v) deoxycholate, 0.1% (w/v) SDS, 50 mM Tris-HCl pH 8.0, 150 mM NaCl, 1 mM β-mercaptoethanol, 100 μM PMSF, 1 mM DTT) supplemented with protease inhibitor cocktail (Roche). Protein concentration was measured using the Pierce BCA assay kit (Thermo Scientific). Briefly, 20 μg protein lysate was fractionated on a 10% (w/v) SDS-PAGE gel and transferred onto a Protran nitrocellulose membrane (Whatman). All primary and secondary antibodies used in this study were previously described [13–15].

### Statistical analysis

All data were analysed with Microsoft Office Excel and Student’s t-test (two tailed) or ONE-WAY ANOVA SPSS (IBM).

## Results

**Fig 1:**
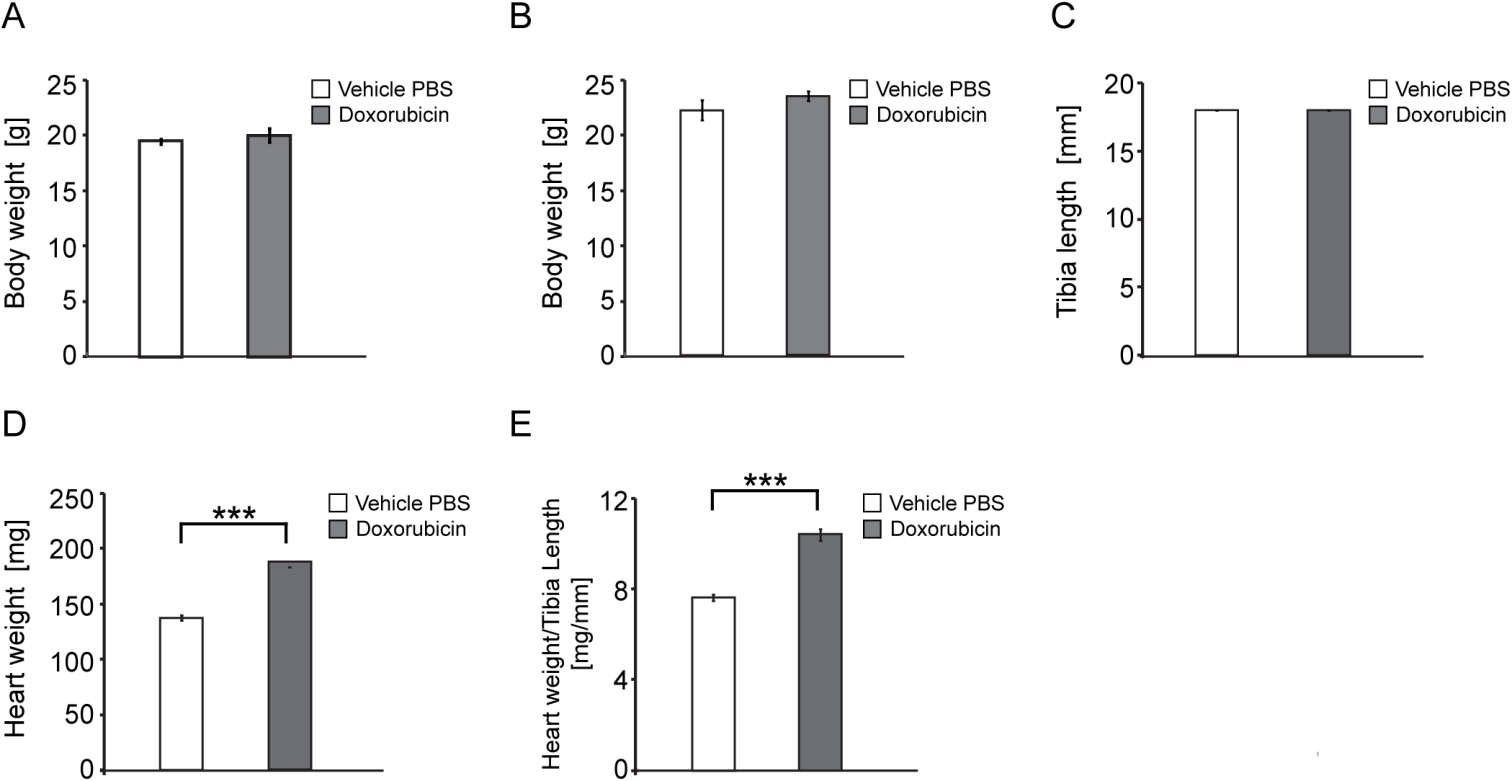
Morphometric analysis of doxorubicin-treated mice. (A) Body weight at 10 weeks of age, prior to implantation of the Alzet pumps. (B) Body weight at the end of the trial (12 weeks of age) (C) tibia length (D) heart weight (E) heart weight to tibia length index. All values are mean ± SEM (*n* = 6 WT PBS, *n* = 6 WT doxorubicin), One-way ANOVA with Bonferroni *post-hoc* test: *p < 0.05, **p < 0.01, ***p < 0.001.

We tested the hypothesis that chronic infusion of a relatively low dose of doxorubicin can induce adverse cardiac remodelling in murine hearts. We administered doxorubicin to wild type (WT) female mice, from 10 weeks of age, for two weeks. Both groups (PBS vehicle and doxorubicin) had comparable body weights at both the start (Fig 1A) and at the end of the trial (Fig 1B). The groups also had comparable tibia lengths at 12 weeks of age (Fig 1C), indicating that there were no gross differences in growth. However, chronic treatment with doxorubicin led to a significant increase in heart weight (Fig 1D). Consequently, the HW/TL index (Heart Weight to Tibia Length) was significantly increased in the WT doxorubicin group, in comparison to the WT vehicle (PBS) group (Fig 1E). We did not observe any general adverse effects that are common in humans, such as eye or skin irritation, or hair loss. Local toxicity or vesicant effects of doxorubicin (tissue damage from escaping out of the vein) do not occur with Alzet pumps and so were also absent. Overall, we conclude from this morphometric analysis that doxorubicin treatment at low dose (15 μg/g infusion) was cardiotoxic and led to a cardiomyopathy that is similar to that previously described for a higher cumulative dose of doxorubicin (24 μg/g) [16].

**Fig 2:**
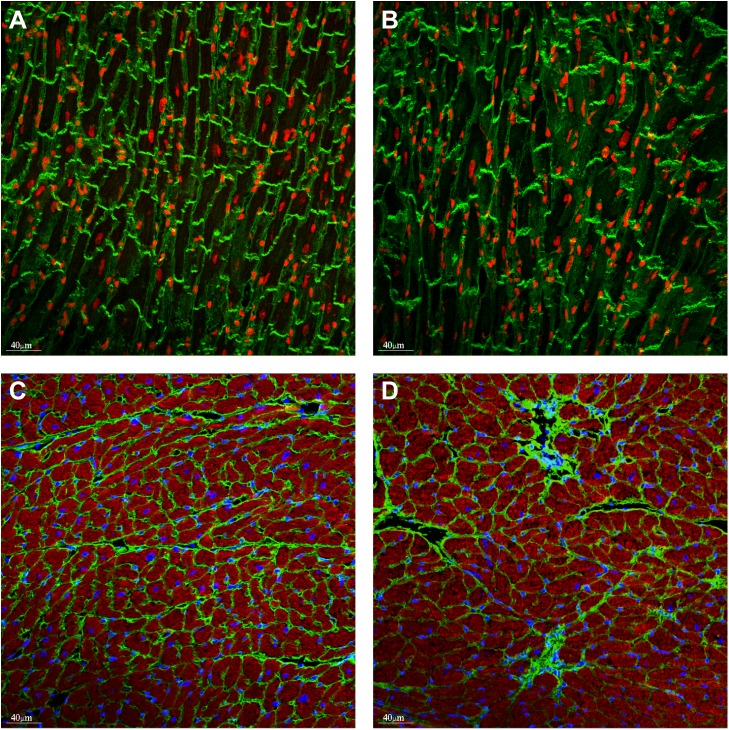
Gross cardiac morphology of hearts treated with doxorubicin. Representative vinculin staining (green) in WT (PBS) mice (A) and WT doxorubicin (B). Nuclei (red) were visualized with draq5. Replacement fibrosis was detected in the doxorubicin treated hearts (D) but not in vehicle hearts (C), visualising with the anti-collagen VI antibody (green). Nuclei (blue) were visualised with DAPI, (E, F).

To further gain insights into doxorubicin-related toxicity, we performed immunohistochemistry on these hearts, in order to visualise the gross morphologies of cardiomyocytes (Fig 2A and 2B). Vinculin staining indicated that doxorubicin treatment causes a disarray of cardiomyocytes. This was accompanied by a replacement fibrosis, as judged based on collagen VI staining (Fig 2C and 2D).

**Fig 3:**
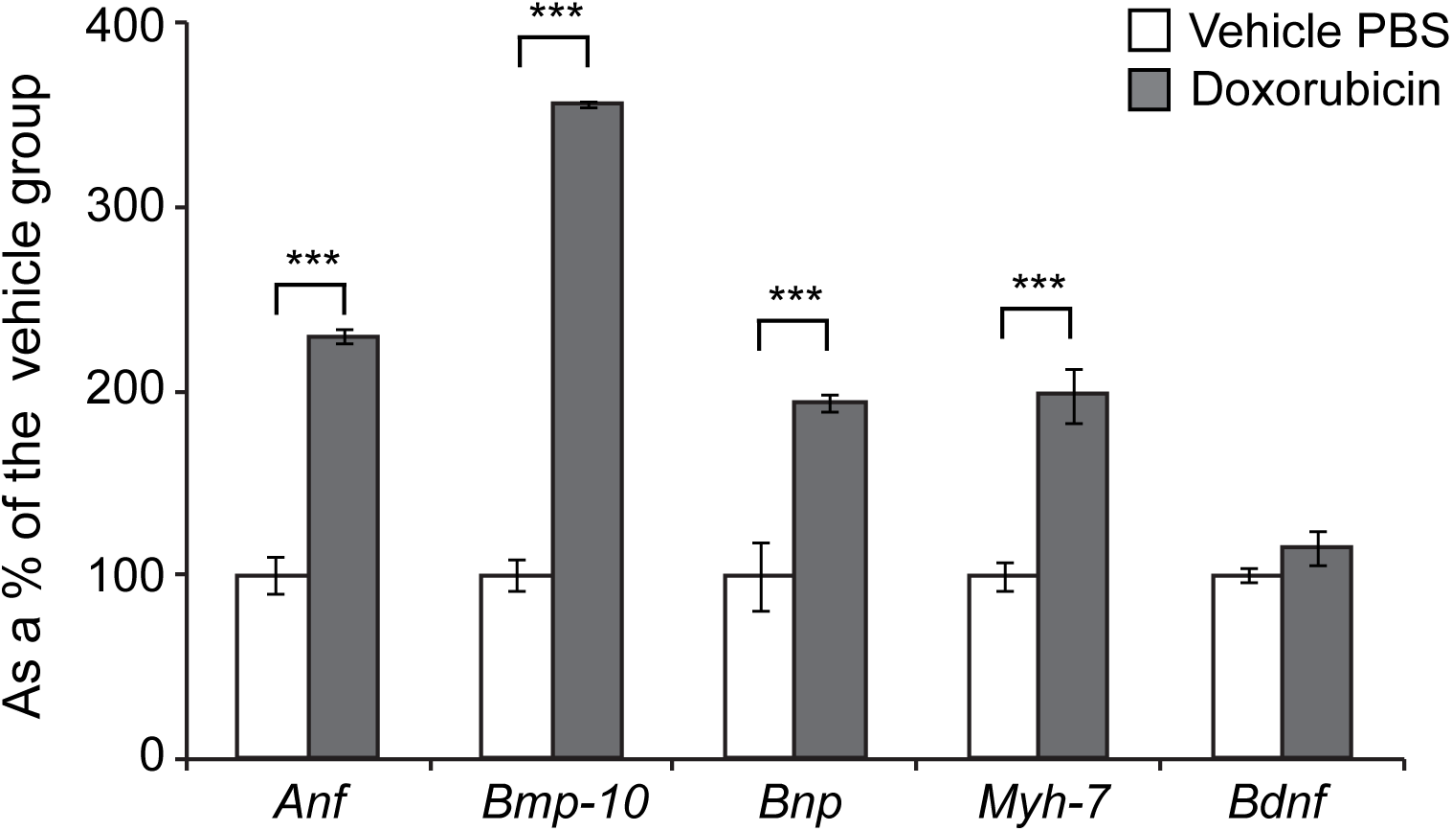
Re-activation of foetal gene markers in hearts treated with doxorubicin. *Anp* (atrial natriuretic peptide), *Bmp-10* (brain morphogentic protein 10), *Bnp* (brain natriuretic protein), and *Myh-7* (myosin heavy chain beta) were elevated in the hearts treated with doxorubicin. *Bdnf* (brain derived neurotophic factor) mRNA remained unchanged. All Taqman qPCR values were normalized to the geometric mean of three housekeeping genes: *Actb*, *Cyc1* and *Gapdh*. Error bars are SEM (n = 6). One-way ANOVA with Bonferroni *post-hoc* test: *p < 0.05, **p < 0.01; ***p < 0.001.

Many types of pathological changes in the heart are associated with a reactivation of the foetal gene programme [17]. We therefore assessed the expression levels of genes known to be changed as a consequence of cardiac hypertrophy or dilated cardiomyopathy (DCM) [12,17]. We found *Anp* (atrial natriuretic peptide) and *Bnp* (brain natriuretic peptide) to be up-regulated in doxorubicin-treated mice, in comparison to their respective vehicle groups (Fig 3). Similarly, we found that the transcripts of two other well-known reactivated foetally-expressed genes, namely *Bmp-10* (bone morphogenetic protein 10) and *Myh-7* (myosin heavy chain beta), were significantly up-regulated in the WT doxorubicin-treated mice (Fig 3). On the other hand, *Bdnf* (brain derived neutrophic factor) is a well-studied gene that has been linked to contractile dysfunction in the heart [18]. Surprisingly, we found that doxorubicin treatment does not alter the transcript level of *Bdnf* (Fig 3), indicating that the damage response is distinct from this pathway.

**Fig 4:**
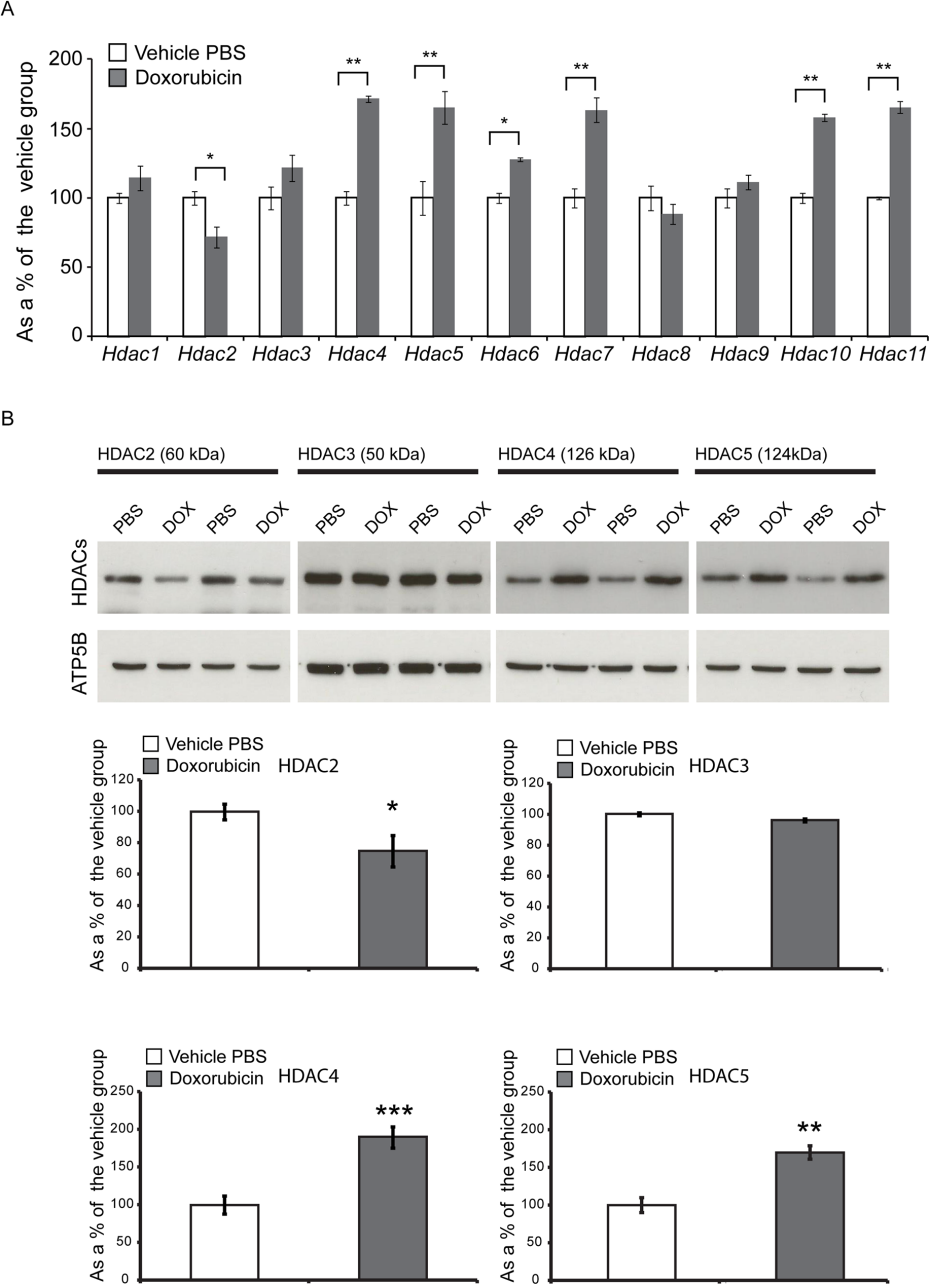
Chronic administration of doxorubicin leads to a significant transcriptional deregulation of *Hdacs*. (A) Transcript levels of *Hdac4*, *Hdac5*, *Hdac6*, *Hdac7*, *Hdac10* and *Hdac11* were increased, while *Hdac2* mRNA was significantly reduced, in the hearts of WT mice treated with doxorubicin. All Taqman qPCR values were normalized to the geometric mean of three housekeeping genes: *Actb*, *Cyc1* and *Gapdh*. Error bars are ±SEM (n = 6). One-way ANOVA with Bonferroni *post-hoc* test: *p < 0.05, **p < 0.01; ***p < 0.001. (B) Representative western immunoblots of 20 μg of heart homogenates from WT doxorubicin and vehicle-treated mice (PBS). Protein levels of HDAC2 are significantly down-regulated; HDAC3 remains unchanged; HDAC4 and HDAC5 are significantly up-regulated. Relative expression levels of HDACs were obtained by normalisation to ATP5b in densitometry; values are mean ± SEM (*n* = 6). Student’s *t* test: *p < 0.05, **p < 0.01, ***p < 0.001.

Typically, the heart responds to pathological stresses or processes by remodelling in a manner that is associated with changes in epigenetic marks and recent studies suggest a key role for histone deacetylases (HDACs) in the control of pathological cardiac remodelling [19–21]. Hence, we sought to profile the transcription of 11 *Hdacs* upon chronic treatment with doxorubicin. We found that treatment leads to a significant down-regulation of *Hdac2* mRNA and that this was the only member of the class I HDACs to be deregulated (Fig 4A). Among class II a and b, we found a significant up-regulation of *Hdac4*, *Hdac5*, *Hdac6*, *Hdac7* and *Hdac10*, while transcript levels of *Hdac9* remained un-changed (Fig 4A). We also noticed that *Hdac11* (the sole member of class IV) was significantly up-regulated in the treated murine hearts, in comparison to their WT littermates (Fig 4A). Next, we investigated whether chronic doxorubicin treatment can affect the protein levels of deregulated HDACs on the transcriptional level. We previously screened a number of commercially-available antibodies raised against HDAC enzymes that were suitable and specific for immunodetection in mouse tissues [13–15]. Using western blotting, we found that doxorubicin had no effect on the protein level of HDAC3 in the heart lysates of WT mice, in comparison to their vehicle group (Fig 4B). However, chronic doxorubicin treatment led to a significant down-regulation of HDAC2 and a significant up-regulation (approximately 2-fold) of HDAC4 and HDAC5 protein levels (Fig 4B).

Overall, this confirms that deregulated transcripts of *Hdacs* can also result in changes at the protein level. Unfortunately, due to the lack of availability of commercial, validated antibodies against HDAC6, HDAC7, HDAC10 and HDAC11, we were not able to assay the protein levels of this subset in doxorubicin-treated murine hearts.

It is believed that given the large body of literature on HDACs in cardiomyopathy, one may conclude that chronic doxorubicin infusion likely exerts its cardiotoxic properties through an epigenetic mechanism related to changes in the transcriptional signature of histone deacetylases. Alternatively, since it is mainly class II HDACs that have been identified here as potential molecular targets for doxorubicin-induced cardiomyopathy, it is very likely that such mechanisms are linked to non-histone actions of class II HDACs, including their well-described properties as transcriptional repressors; for a review see [22,23].

## Discussion

Anthracyclines such as doxorubicin (adriamycin) are widely-used anticancer drugs, with a proven therapeutic potential in many haematological cancers and solid malignancies [24]. Despite the beneficial effect of doxorubicin in cancer, it is well-established that this drug causes a severe cardiomyopathy, and heart failure is observed in doxorubicin-treated cancer patients [25]. Although the precise mechanism of doxorubicin's cardiotoxicity remains largely unknown, there have been a number of studies suggesting that doxorubicin might act through oxidative stress, including superoxide radical production, mitochondrial DNA damage, or even an imbalance in calcium or iron homeostasis; for a review see [25]. However, until now, little has been known about whether doxorubicin changes epigenetic regulation in the heart. Hence, we sought to establish whether there were any changes in the transcriptional signature of histone deacetylases (Hdacs), using a well-established mouse model with chronic administration of doxorubicin [26,27].

For the first time, we can report that the following *Hdacs* are significantly deregulated in doxorubicin-treated murine hearts: *Hdac2*, *Hdac4*, *Hdac5*, *Hdac6*, *Hdac7*, *Hdac10* and *Hdac11*. Interestingly, the majority of altered *Hdac* transcripts belong to the class II (a and b) and IV sub-families. Class IIa HDACs have already been linked to the hypertrophy of cardiomyocytes, likely through their repressive propensities via the MEF2 family of transcription factors [28]. Interestingly, the loss of miR-22 (which targets HDAC4) led to the development of dilated cardiomyopathy under stress conditions [29]. HDAC7 has been showed to control endothelial growth via its interaction with beta-catenin, a mechanism that is independent of its enzymatic domain [30]. While HDAC2-deficient mice showed a partial lethality due to early myocardial defects [31], there are no data available about the function of HDAC10 and 11 in the heart. In fact, *Hdac6* has already been identified as a molecular target in a cardiomyopathy mouse model based on the accumulation of misfolded proteins [32]. This is in contrast to the *Hdac* transcriptional profile in hypertrophic hearts, where class I and IIb were mainly deregulated, such as in the chronic isoproterenol mouse model [12]. Therefore doxorubicin-induced cardiomyopathy is mediated through a different subset of *Hdac* members than those responding to hypertrophic signals.

In this study we did not explore the dose-dependency of the HDAC transcriptional alterations with doxorubicin. Although it would be interesting to test whether lower doses of doxorubicin would still affect the transcriptional profile of HDACs, this would likely lead to a reduction in the therapeutic effectiveness of doxorubicin towards its primary target–cancer cells–and so would not solve the problem by itself. Nonetheless, the distinctive alterations in *Hdacs* raise intriguing new possibilities for therapeutic approaches that might ameliorate doxorubicin toxicity. Previous studies have demonstrated the efficiency of HDAC inhibitors in reducing cardiac hypertrophy under pathological conditions [33,34] and in attenuating structural remodelling after myocardial infarction [35]. In fact, a detailed map of chromatin modification caused by two well-used pan-HDAC inhibitors, namely TSA and SAHA, has been described in a human aortic endothelial cell model [36]. Moreover, recent clinical studies favour dexrazoxane (a topoisomerase-2 inhibitor) as a cardioprotective agent in doxorubicin-induced cardiomyopathy (for a review see [37]). Interestingly, there are reports that in cancer cell lines HDAC inhibitors might facilitate selective degradation of topoisomerase-2 [38] and act in a similar way to dexrazoxane. Our data imply that it would be worthwhile to elucidate the apparently different pathways involved in doxorubicin-induced transcriptional deregulation of *Hdacs*. By understanding this complex cardiac pathological response better, it may perhaps be possible to intervene pharmacologically. For example, specific selective HDAC inhibitors could target the cytotoxic effect of doxorubicin in the heart. It is well established that some pan-HDAC inhibitors are beneficial in a model of cardiac hypertrophy [34], and may attenuate structural remodelling after myocardial infarction [35]. Alternatively, suberoylanilide hydroxamic acid (SAHA; an inhibitor of Class I and II HDACs) reduced protein aggregates in cardiomyocytes and led to substantial improvement in cardiac function [32]. Our study would suggest that lowering the activities of HDAC4, 5, 7, 10 and 11 might be beneficial in managing doxorubicin cardiotoxicity. However, class IIa HDACs possess a single amino acid exchange (tyrosine in class I HDACs versus a histidine in class IIa HDACs) that is responsible for their reduced enzymatic activity [39]. In fact, a reverse His-to-Tyr mutation (HDAC4 His-976-Tyr) led to a remarkable 1,000-fold increase in the histone deacetylase activity [39]. This is in line with our recently published immunoaffinity-based AcetylScan proteomic screen; although this identified many proteins that are known to be modified by acetylation, in the absence of HDAC4 there was no effect on the acetylation profile of the murine neonate brain [40]. This further reinforces that HDAC4 is not a true deacetylase. Since HDAC4 shares a high homology with other class IIa members, it is highly likely that these other members are also not true deacetylase enzymes. Hence, developing specific inhibitors that alter class IIa activities that are not related to the deacetylase domain might be a better therapeutic strategy for doxorubicin-induced cardiomyopathy.

It is well known that cardiac pathological epigenetic remodelling plays a pivotal role in disease and aging. For example, HDACs control crucial pathological events in the heart, such as hypertrophy [33,41], fibrosis [42], contractility [43], and energy metabolism [44]. In fact, an increased global activity of HDACs has been associated with various adverse cardiac conditions, including hypertrophy [12,34], cardiac ischemia-reperfusion injury [45] and Huntington’s disease related cardiomyopathy [12,14]. Our data also imply that the epigenetic alterations observed with low-dose doxorubicin are indeed resulting in remodelling of gene expression. For example, we observed a re-activation of foetal genes such as *Anf*, *Bnf* and *Bmp-10* and *Myh-7*, which is characteristic for many kinds of heart pathology. However, the profile remained unchanged for levels of *Bdnf*, a gene that has been associated with the contractile dysfunction in the heart [18], indicating that doxorubicin cardiotoxicity does not affect this pathway under these conditions. Again, this is an indication that the genetic and epigenetic pathways regulating this response require further characterisation, in order to tailor cardioprotective therapies during doxorubicin treatment. One might also assume that more specific HDAC inhibitors i.e. targeting only class IIa and b could be more efficient against doxorubicin-induced cardiomyopathy.

Overall, in this study we aimed to verify whether there were any changes in epigenetic regulation in response to doxorubicin in the murine heart. The observation that there are distinct changes, even under conditions which are generally considered to be cardioprotective, suggests that new approaches to reducing doxorubicin toxicity should now be explored.
